# Fatty acid composition of erythrocytes in multiple organ dysfunction syndrome

**DOI:** 10.1186/cc14105

**Published:** 2015-03-16

**Authors:** A Osipenko, A Marochkov

**Affiliations:** 1 A. Kuleshov Mogilev State University, Mogilev, Belarus; 2 Mogilev Regional Hospital, Mogilev, Belarus

## Introduction

Change in fatty acid composition of erythrocytes and blood plasma in cases of various pathological conditions is evidence of lipid metabolism disorder and can indicate the reasons for and the degree of these disorders [1]. The aim of this study was to assess the FA composition of plasma and erythrocytes in patients with multiple organ dysfunction syndrome (MODS).

## Methods

The objects of study were 19 people with MODS (37.6 ± 8.3 years) of various etiologies. The blood of 17 healthy volunteers aged 38.4 ± 3.3 years served as control. The FA analysis was conducted using capillary gas-liquid chromatography. Quantitative evaluation of individual FA content was made as a mass percentage of their total (C_14:0 _to C_22:6_). Statistical analysis was performed using the Mann-Whitney *U *test (*P *< 0.05).

## Results

Our data indicate that changes in blood plasma FA composition in patients with MODS are mainly caused by activation of lipolysis in fat depots and are accompanied by an increase of monounsaturated fatty acids, a decrease in saturated stearic acid and polyunsaturated fatty acids in the ratio. In conditions of increased level of monounsaturated palmitoleic (C_16:1_) and oleic (C_18:1_) FA in blood plasma (2.53 ± 0.40% vs. 1.55 ± 0.29%, *P *< 0.001 and 25.18 ± 2.15% vs. 16.55 ± 1.17%, *P *< 0.001, respectively), only the level of palmitoleic (C_16:1_) acid is increased in erythrocytes (0.56 ± 0.12% vs. 0.16 ± 0.12%, *P *< 0.001). Despite the high content of oleic (C_18:1_) acid in blood plasma in case of MODS, in erythrocytes its relative level is not changed as compared with the control group. The disorder of lipid composition constancy in erythrocyte membranes is also manifested by change in the content of saturated palmitic (C_16:0_) and polyunsaturated linoleic (C_18:2_) fatty acids. In the test group of patients, as compared with the control group there was an elevated level (27.12 ± 0.78% vs. 25.80 ±0.77%, *P *< 0.05) of saturated palmitic (C_16:0_) acid combined with the reduced (11.46 ± 0.52% vs. 13.95 ± 1.09%, *P *< 0.001) level of linoleic (C_18:2_) acid.

## Conclusion

The changes revealed in fatty acid composition of erythrocytes may indicate systemic modifications of cell membranes in MODS.
